# Nut consumption and the prevalence and severity of non-alcoholic fatty liver disease

**DOI:** 10.1371/journal.pone.0244514

**Published:** 2020-12-31

**Authors:** Georg Semmler, Sebastian Bachmayer, Sarah Wernly, Bernhard Wernly, David Niederseer, Ursula Huber-Schönauer, Felix Stickel, Elmar Aigner, Christian Datz

**Affiliations:** 1 Department of Internal Medicine, General Hospital Oberndorf, Teaching Hospital of the Paracelsus Medical University Salzburg, Oberndorf, Salzburg, Austria; 2 Second Department of Medicine, Paracelsus Medical University Salzburg, Salzburg, Austria; 3 Department of Cardiology, University Hospital Zurich, Zurich, Switzerland; 4 Department of Gastroenterology and Hepatology, University Hospital Zurich, Zurich, Switzerland; 5 First Department of Medicine, Paracelsus Medical University Salzburg, Salzburg, Austria; Medizinische Fakultat der RWTH Aachen, GERMANY

## Abstract

**Background:**

Nut consumption has been associated with reduced inflammation, insulin resistance, and oxidative stress. However, the influence on the prevalence and severity of non-alcoholic fatty liver disease (NAFLD) has yet to be evaluated.

**Methods:**

4655 subjects were included as part of a colorectal carcinoma screening program (SAKKOPI) between 07/2010 and 07/2019 and analyzed 2020. Patients were characterized using biochemical and metabolic parameters, as well as a detailed questionnaire on dietary habits. The diagnosis of NAFLD was established using abdominal ultrasound. Consumption of nuts was graded as: no consumption or <1 time/week, 1–6 times/week, 1 time/day and ≥2 times/day.

**Results:**

Mean age was 58.5±9.8years with a mean BMI of 26.5±4.7kg/m^2^. 2058 (44.2%) patients suffered from the metabolic syndrome, 2407 (51.6%) had arterial hypertension, 2287 (49.1%) showed prediabetes/diabetes, 1854 (39.4%) had dyslipidemia and 1984 patients (43.5%) were diagnosed with NAFLD. Prevalence of metabolic syndrome (1219 [48.7%] vs. 605 [40.2%] vs. 189 [37.4%] vs. 45 [31.7%], p<0.001) and NALFD (1184 [48.1%] vs. 594 [40.7%] vs. 158 [31.7%] vs. 48 [34.0%], p<0.001). On multivariable logistic regression analysis adjusting for potential confounders and dietary patterns, nut consumption ≥1time/day was inversely associated with NAFLD in the overall cohort (adjusted Odds ratio[aOR]: 0.719 [95%CI:0.558–0.926], p = 0.011). However, following subgroup analysis, this inverse association was only confirmed in male patients (aOR: 0.589 [95%CI: 0.411–0.844], p = 0.004) but not in females (aOR: 0.886 [95%CI: 0.616–1.275], p = 0.515). Moreover, patients who consumed nuts 1–6 times/week had a significantly lower prevalence of advanced fibrosis (Fib-4 score >2.67: aOR: 0.551 [95%CI: 0.338–0.898], p = 0.017; Forns-Index >6.9: aOR: 0.585 [95%CI: 0.402–0.850], p = 0.005).

**Conclusions:**

Nut consumption might exert beneficial effects on the prevalence of NAFLD in males. The negative association with advanced fibrosis warrants further investigation.

## Introduction

Non-alcoholic fatty liver disease (NAFLD) is regarded the emerging complex chronic liver disease of the 21^st^ century, being significantly associated with increased cardiovascular and liver-related risk [1–3]. Despite the increasing prevalence of NAFLD, no medical therapy has been established. Thus, lifestyle and dietary interventions remain the cornerstone of treatment in these patients.

Nuts are nutrient-dense food containing bioactive compounds regarded beneficial for health: vegetable protein, unsaturated fatty acids, fiber, minerals, vitamins, tocopherols, phytosterols, and polyphenols [4]. However, each nut type has a unique composition and different amounts led to different health outcomes in interventional studies [5]. Importantly, most data supporting a beneficial effect of nuts exist for almonds, walnuts, pistachios, and peanuts, followed other nut types [5]. For instance, two randomized controlled trials could confirm the lipid-lowering effect of 10/60g almonds/day in patients with coronary artery disease or type 2 diabetes mellitus (T2DM) [6, 7]. In large population-based studies not differentiating between nut types [4], their consumption has been associated with reduced inflammation [8, 9] and insulin resistance (IR) [10]. Moreover, a large body of evidence exists for the protective effect of nuts on T2DM [11, 12], metabolic syndrome (MetS) [12–14], obesity [10, 12, 13] and arterial hypertension [15]. Apart from these beneficial effects on aspects of the MetS, their role in chronic liver disease and especially NAFLD merits further investigation since inflammation, oxidative stress and IR are considered key drivers of NAFLD [16–18]. Recently, a Chinese study reported a significantly lower prevalence of NAFLD in patients consuming nuts ≥4 times/week [19]. However, another Chinese study confirmed this inverse association of NAFLD and nut consumption only in a small subgroup of men when consuming ≥8.86g/day [20] while data from Caucasian subjects addressing this topic is scarce. Therefore, we aimed to further elucidate the role of nut consumption on the prevalence and severity of NAFLD.

## Materials & methods

### Patients and design

4655 individuals participating in a screening program for colorectal cancer (SAKKOPI) between 07/2010 and 07/2019 were included in this analysis. Only patients with established liver disease (i.e. viral hepatitis, autoimmune hepatitis, hereditary liver diseases) or with consumption of a significant amount of alcohol (≥2 drinks/day for females and ≥3 drinks/day for males) were excluded from the analysis. All other patients without established liver disease were included portraying a representative sample of the general population. Patients were carefully characterized including laboratory and clinical data, and completed a detailed questionnaire on lifestyle and dietary habits. The study design and details of the clinical and biochemical work-up of included subjects have been reported previously [21]. The study was approved by the “Ethikkommission für das Bundesland Salzburg” (approval no. 415-E/1262/2-2010), and all patients gave written informed consent to participate.

### Dietary assessment

Dietary data were collected using a food-frequency questionnaire (FFQ) which consisted of 69-items and was conducted in line with the questionnaire used in the SAPHIR [22], as well as the EPIC study [23–25]. Patients completed the FFQ self-administered prior to all examinations. Data on alcohol consumption, intake of fast-food, vegetables, fruits, sweets, red and processed meat, white meat, fish, coffee and consumption of sugar-sweetened beverages (SSB) and nuts were assessed. Specifically, nut consumption was reported semiquantitatively as no consumption or <1 time (corresponding to ~10g) per week, 1–6 times per week, 1 time per day and ≥2 times per day. Different types of nuts were not specified, and legume seeds were included according to the interpretation of the patient. In order to quantify healthy eating patterns, an adopted version of the “Alternate Healthy Eating Index” (AHEI-2010) was calculated in accordance with the FFQ being available [26].

### Definitions

The diagnosis of NAFLD was established using ultrasound being performed by experienced operators. The liver was considered”normal” if the echogenicity was homogenous and similar or slightly higher than that of the renal parenchyma. Portal veins and diaphragm had to be visualized. The liver was considered as”fatty liver” when areas of significant increased echogenicity in relation to the renal parenchyma were found. The severity of sonographic steatosis was not graded [21]. Advanced fibrosis (≥F3) was assessed using Fib-4 score, NAFLD fibrosis score (NFS) and Forns-Index [27, 28]. Cut-offs for advanced fibrosis were determined as previously described: >2.67 [27, 29] for Fib-4 score, >0.676 for NFS [30] and >6.9 for Forns-Index [31]. Metabolic characterization included an oral glucose tolerance test (OGTT) as well as measurement of fasting blood glucose and insulin measurement to assess IR. T2DM was defined as either blood glucose level of ≥200mg/dl after 2 hours following oral glucose tolerance test (OGTT), fasting blood glucose (FBG) ≥125mg/dl, HbA1c ≥6.5%, or previously prescribed antidiabetic medication including insulin. Impaired fasting glucose (IFG) was defined as FBG 100-124mg/dl in non-diabetic individuals. Impaired glucose tolerance (IGT) was defined as a blood glucose of 140-199mg/dl after 2 hours following OGTT in non-diabetic individuals. Prediabetes was defined as IFG or IGT in non-diabetic individuals. Dysglycemia was defined as presence of either prediabetes or T2DM. Components of the MetS were defined according to the revised definition of the International Diabetes Federation (2005) [32]. Visceral obesity was defined as waist circumference (WC) ≥80cm for women and ≥94cm for men. Hypertension was defined as a blood pressure (BP) ≥130/85mmHg or previous prescription of any antihypertensive drug. Dyslipidemia was defined as high-density lipoprotein cholesterol (HDL-C) <40mg/dl in males or <50mg/dl in females, triglycerides ≥150mg/dl or previously prescribed lipid lowering drugs. The MetS was defined as at least three of the following components: Visceral adiposity, hypertension, FBG ≥100mg/dl or antidiabetic therapy, HDL-C <40mg/dl in males or <50mg/dl in females or antilipidemic drugs, triglycerides ≥150mg/dl or antilipidemic drugs. Of note, levels of systolic BP, FBG, blood glucose following OGTT, HbA1c, HOMA-IR, triglycerides, cholesterol, HDL-C and low-density lipoprotein cholesterol (LDL-C) were only considered for comparison in the absence of specific medication. Chronic coronary syndrome (CCS) was defined as history of myocardial infarction, coronary artery disease, coronary artery bypass graft or coronary stent.

### Statistical analyses

Statistical analyses were performed using IBM SPSS Statistics 26 (SPSS Inc., Armonk, New York, USA) and GraphPad Prism 8 (GraphPad Software, La Jolla, California, USA) in 2020. Continuous variables were reported as mean ± standard deviation or median (interquartile range [IQR]) depending on their distribution, while categorical variables were shown as numbers and proportions of patients (%). Comparisons of continuous variables were performed using Student’s t-test, Mann-Whitney U test or Kruskal-Wallis test, as applicable. Group comparisons were performed applying Chi-squared test. Logistic regression analysis was performed to investigate the associations of nut consumption on the presence of NAFLD and the presence of advanced fibrosis, using the group with the lowest nut consumption as reference category. Since the number of individuals in group 4 was too limited to draw firm conclusions, groups 3 and 4 were combined for logistic regression analyses. P-values were calculated for each group compared to the reference group, as well as P-values for linear trends. Multivariable models were adjusted for potential confounders including sex, age and BMI, MetS, and dietary parameters such as alcohol consumption, intake of fast-food, vegetables, fruits, sweets, red and processed meat, white meat, fish, coffee and consumption of SSB, as previously described [19, 20]. Analyses on Fib-4 score and Forns-Index were not adjusted for age since this variable is included in these indices. Subgroup analyses were performed in patients with NAFLD, male and female individuals. A two-sided p-value ≤0.05 was considered as statistically significant.

## Results

### Patient characteristics

Overall, 4655 individuals were included with a slight preponderance of males (n = 2395, 51.5%), and a mean age of 58.5±9.8. 2058 patients (44.2%) suffered from the MetS with 3278 (76.9%) suffering from visceral obesity, 2407 (51.6%) from hypertension, 2287 (49.1%) from T2DM or prediabetes, and 1854 (39.8%) from dyslipidemia. Additionally, 280 (6.0%) individuals had previously diagnosed CCS, 152 (3.3%) peripheral arterial disease (PAD) and 148 (3.2%) had previously suffered from stroke. 2502 patients (53.7%) did not consume nuts or consumed nuts <1 time/week, 1506 patients (32.4%) consumed nuts 1–6 times/week, 505 patients (10.8%) consumed nuts 1 time/day (once daily) and 142 patients (3.1%) ≥2 times/day (at least twice daily).

### Nut consumption, metabolic syndrome and cardiovascular health

The prevalence of the MetS declined with more frequent nut consumption (48.7% vs. 40.2% vs. 37.4% vs. 31.7%, p<0.001). Importantly, this trend was also observed for all singular components (Table 1). Similarly, the proportion of patients suffering from cardiovascular diseases decreased with increasing nut consumption: 7.5% vs. 4.4% vs. 4.0% vs. 3.5% for CCS (p<0.001), 4.3% vs. 2.0% vs. 2.6% vs. 2.1% for PAD (p = 0.001) and 4.0% vs. 2.1% vs. 2.8% vs. 1.4% for stroke (p = 0.005).

**Table 1:** Patient characteristics in the overall cohort and compared among patients grouped according to their frequency of nut consumption.

Patient characteristics		All patients, n = 4655	<1 time/week, n = 2502	1–6 times/week, n = 1506	1 time/day, n = 505	≥2 times/day, n = 142	*P* value
Age, years		58.5±9.8	58.8±10.2	57.6±9.2	59.8±8.7	58.2±8.8	**<0.001**
Male sex		2395 (51.5%)	1360 (54.4%)	766 (50.9%)	209 (41.4%)	60 (42.3%)	**<0.001**
Obesity		1068 (22.9%)	661 (26.5%)	302 (20.1%)	77 (15.2%)	29 (19.7%)	**<0.001**
	BMI, kg/mg^2^	26.5±4.7	27.6±4.9	26.7±4.5	25.9±4.2	25.9±4.5	**<0.001**
Visceral obesity*		3278 (76.9%)	1814 (79.9%)	1050 (75.1%)	331 (71.3%)	83 (62.4%)	**<0.001**
	WC, cm*	96.3±13.3	98.1±13.6	95.2±12.6	92.5±12.3	92.2±14.2	**<0.001**
Metabolic syndrome		2058 (44.2%)	1219 (48.7%)	605 (40.2%)	189 (37.4%)	45 (31.7%)	**<0.001**
Hypertension		2407 (51.6%)	1776 (71.0%)	972 (64.5%)	341 (67.5%)	94 (66.2%)	**<0.001**
T2DM/prediabetes		2287 (49.1%)	1334 (53.3%)	679 (45.1%)	226 (44.8%)	48 (33.8%)	**<0.001**
T2DM		638 (13.7%)	422 (16.9%)	151 (10.0%)	47 (9.3%)	18 (12.7%)	**<0.001**
Prediabetes		1649 (35.4%)	912 (36.5%)	528 (35.1%)	179 (35.4%)	30 (21.1%)	**0.003**
	IFG	1365 (29.3%)	753 (30.1%)	446 (29.6%)	139 (27.5%)	27 (19.0%)	**0.001**
	IGT	638 (13.7%)	362 (14.5%)	193 (12.8%)	76 (15.0%)	7 (4.9%)	**<0.001**
	FBG, mg/dl	98±20	99±15	98±27	96±11	96±18	**0.008**
	OGTT after 2h, mg/dl	122±37	125±39	119±33	122±33	119±39	**<0.001**
	HbA1c, %	5.5±0.4	5.6±0.5	5.5. ±0.4	5.5±0.4	5.5±0.4	**<0.001**
	HOMA-IR, points	1.67 (1.11–2.62)	1.79 (1.15–2.81)	1.63 (1.13–2.53)	1.46 (1.00–2.30)	1.46 (0.90–2.30)	**<0.001**
Dyslipidemia		1854 (39.8%)	1083 (43.3%)	553 (36.7%)	174 (14.5%)	44 (31.0%)	**<0.001**
	Triglycerides, mg/dl	102 (76–142)	106 (78–148)	101 (74–140)	93 (72–129)	88 (67–126)	**<0.001**
Hypertriglyceridemia		879 (18.9%)	507 (20.3%)	281 (18.7%)	72 (14.3%)	19 (13.4%)	**<0.001**
	Cholesterol, mg/dl	224±41	223±43	226±40	225±39	222±41	0.333
Hypercholesterinemia		2857 (61.4%)	1463 (58.5%)	991 (65.8%)	315 (62.4%)	88 (62.0%)	**0.040**
	HDL-C, mg/dl	60±17	59±17	60±18	62±17	64±17	**<0.001**
	LDL-C, mg/dl	145±37	145±38	147±37	145±36	140±37	0.244
CCS		280 (6.0%)	188 (7.5%)	67 (4.4%)	20 (4.0%)	5 (3.5%)	**<0.001**
PAD		152 (3.3%)	106 (4.3%)	30 (2.0%)	13 (2.6%)	3 (2.1%)	**0.001**
Stroke		148 (3.2%)	100 (4.0%)	32 (2.1%)	14 (2.8%)	2 (1.4%)	**0.005**

* Data available in 4265 patients.

Abbreviations: BMI–body mass index; CCS–chronic coronary syndrome; FBG–fasting blood glucose; HOMA-IR–Homeostasis Model Assessment for Insulin Resistance; IFG–impaired fasting glucose; IGT–impaired glucose tolerance; OGTT–oral glucose tolerance test; PAD–peripheral artery disease; T2DM–type 2 diabetes mellitus; WC–waist circumference.

Dietary patterns including consumption of alcohol, fast-food, fruits, vegetables, sweets, coffee, SSBs, red meat and fish were significantly different among these groups with less frequent fast-food consumption and consumption of red or processed meat and a higher consumption of fruits, vegetables and coffee in patients with more frequent nut consumption (Table 2). Consecutively, these subjects had higher scores in the adopted version of the AHEI-2010 (42.4±8.7 vs. 49.3±8.2 vs. 57.6±7.8 vs. 60.6±8.2 points, p<0.001).

**Table 2:** Dietary patterns compared among patients grouped according to their frequency of nut consumption.

Patient characteristics	<1 time/week, n = 2502	1–6 times/week, n = 1506	1 time/day, n = 505	≥2 times/day, n = 142	*P* value
Alcohol consumption ≥3 times/week	31.0%	37.5%	29.7%	32.4%	**<0.001**
Fast food ≥1/month	51.0%	57.9%	41.5%	34.8%	**<0.001**
Daily fruit intake	59.5%	71.8%	84.5%	84.1%	**<0.001**
Daily vegetable intake	63.8%	75.4%	86.2%	86.3%	**<0.001**
Daily sweets intake	30.8%	37.3%	41.5%	39.1%	**<0.001**
Daily coffee intake	48.6%	58.4%	53.1%	54.9%	**<0.001**
Daily SSB intake	10.2%	13.9%	10.3%	9.9%	**0.002**
Red meat ≥2days/week	71.3%	65.4%	55.7%	46.6%	**<0.001**
White meat ≥2days/week	32.5%	32.8%	31.9%	27.5%	0.663
Fish ≥1day/week	76.4%	86.8%	83.8%	85.5%	**<0.001**
Adopted AHEI-2010*	42.4±8.7	49.3±8.2	57.6±7.8	60.6±8.2	**<0.001**

* The percentage of trans-Isomers of fatty acids and polyunsaturated fatty acids from total energy intake could not be sufficiently calculated owing to the nature of our FFQ. Therefore, these variables could not be calculated leading to a maximum score of 90 points.

Abbreviations: SSB–sugar-sweetened beverages.

### Nut consumption and NAFLD

The prevalence of NAFLD decreased among nut consumption groups: 1184 patients (48.1%) with nut consumption <1time/week, 594 patients (40.7%) with 1–6 times/week, 158 (31.7%) with 1time/day and 48 patients (34.0%) with ≥2times/day were diagnosed with NAFLD (p<0.001, Table 3). The prevalence of a Fib-4 score of >2.67 indicative for advanced fibrosis was significantly different among groups (3.9% vs. 2.0% vs. 3.2% vs. 3.5%, p = 0.014), concordantly to the prevalence of NFS >0.676 (5.6% vs. 1.9% vs. 3.0% vs. 2.4%, p<0.001) and Forns-Index >6.9 (6.9% vs. 3.4% vs. 4.2% vs. 2.1%, p<0.001). Finally, patients had less elevated levels of gamma-glutamyl transferase (GGT; median GGT/sex-specific upper limit of normal: 0.69 [IQR: 0.46–1.18] vs. 0.64 [0.44–1.08] vs. 0.56 [0.38–0.87] vs. 0.51 [0.37–0.82], p<0.001).

**Table 3:** Distribution of hepatic steatosis as well as non-invasive scores for hepatic fibrosis compared among patients grouped according to their frequency of nut consumption.

Patient characteristics		<1 time/week, n = 2502	1–6 times/week, n = 1506	1 time/day, n = 505	≥2 times/day, n = 142	*P* value
NAFLD*		1184 (48.1%)	594 (40.7%)	158 (31.7%)	48 (34.0%)	**<0.001**
Fib-4 score		1.16 (0.89–1.53)	1.12 (0.88–1.43)	1.17 (0.95–1.58)	1.19 (1.00–1.54)	**<0.001**
	>2.67	96 (3.9%)	30 (2.0%)	16 (3.2%)	5 (3.5%)	**0.014**
	>3.25	58 (2.3%)	20 (1.3%)	10 (2.0%)	1 (0.7%)	0.108
NFS		-1.64 (-2.41-[-0.70])	-1.88 (-2.51-[-1.17])	-1.81 (-2.49-[-0.93])	-1.81 (-2.30-[-0.83])	**<0.001**
	>0.676	64 (5.6%)	17 (1.9%)	9 (3.0%)	2 (2.4%)	**<0.001**
Forns-Index		4.51±1.55	4.29±1.36	4.37±1.37	4.31±1.31	**<0.001**
	>6.9	171 (6.9%)	51 (3.4%)	21 (4.2%)	3 (2.1%)	**<0.001**
ALT/ULN		0.50 (0.38–0.71)	0.50 (0.37–0.69)	0.49 (0.37–0.66)	0.49 (0.37–0.58)	0.055
AST/ULN		0.60 (0.51–0.74)	0.60 (0.49–0.71)	0.57 (0.49–0.71)	0.60 (0.51–0.66)	0.056
GGT/ULN		0.69 (0.46–1.18)	0.64 (0.44–1.08)	0.56 (0.38–0.87)	0.51 (0.37–0.82)	**<0.001**

* Data available in 4563 patients.

ULN was regarded as 50 U/L for ALT and AST in male patients and 35 U/L in female patients.

Abbreviations: ALT–alanine aminotransferase; AST–aspartate aminotransferase; GGT–gamma-glutamyl transferase; NAFLD–non-alcoholic fatty liver disease; NFS–NAFLD fibrosis score; ULN–upper limit of normal.

On multivariable logistic regression analysis adjusting for potential confounders, patients consuming nuts ≥1time/day had a significantly lower risk for NAFLD (aOR: 0.589 [95%CI: 0.558–0.926], p = 0.011) when compared to those within the lowest group (Table 4). However, a linear trend across all groups was observed (p = 0.016). Interestingly, patients consuming nuts 1–6 times/week had a significantly lower odds of advanced fibrosis (Fib-4 score >2.67: aOR: 0.551 [95%CI: 0.338–0.898], p = 0.017; Forns-Index >6.9: aOR: 0.585 [95%CI: 0.402–0.850], p = 0.005) which was more pronounced in the subgroup of NAFLD patients (Fib-4 score >2.67: aOR: 0.275 [95%CI: 0.112–0.678], p = 0.005; Forns-Index >6.9: aOR: 0.384 [95%CI: 0.226–0.653], p<0.001). In order to identify potential reasons for these inverse associations, markers of inflammation were compared among subgroups of nut-consumption. Interestingly, subjects with more frequent nut consumption had significantly lower levels of C-reactive protein concentrations (0.20 [IQR: 0.10–0.40] vs. 0.17 [0.10–0.31] vs. 0.15 [0.08–0.30] vs. 0.11 [0.08–0.27] mg/dl, p<0.001) and ferritin concentrations (134 [71–231] vs. 114 [63–193] vs. 103 [54–178] vs. 89 [42–159] μg/l, p<0.001; S1 Table in S1 File). Of note, when replacing dietary patterns with the adopted AHEI-2010 for regression analyses, there was no independently association with NAFLD (aOR per point: 0.992 [95%CI: 0.984–1.000], p = 0.063).

**Table 4:** Odds Ratio (OR) and 95% confidence interval (95%CI) for NAFLD and advance fibrosis among groups of nut-consumers using binary logistic regression analyses adjusted for potential confounders.

		Overall cohort			NAFLD		
		aOR (95% CI)	*P* value	*P* value for linear trend	aOR (95% CI)	*P* value	*P* value for linear trend
**NAFLD**	**<1 time/week**	reference	reference	**0.016**	-	-	-
	**1–6 times/week**	0.923 (0.770–1.106)	0.384		-	-	-
	**≥1 time/day**	0.719 (0.558–0.926)	**0.011**		-	-	-
**Fib-4 score >2.67**	**<1 time/week**	reference	reference	0.110	reference	reference	0.099
	**1–6 times/week**	0.551 (0.338–0.898)	**0.017**		0.275 (0.112–0.678)	**0.005**	
	**≥1 time/day**	0.780 (0.427–1.423)	0.418		0.844 (0.337–2.110)	0.716	
**Forns-Index >6.9**	**<1 time/week**	reference	reference	**0.002**	reference	reference	**0.001**
	**1–6 times/week**	0.585 (0.402–0.850)	**0.005**		0.384 (0.226–0.653)	**<0.001**	
	**≥1 time/day**	0.533 (0.304–0.934)	**0.028**		0.450 (0.198–1.023)	0.057	

Analyses were based on the overall cohort and NAFLD patients.

Displayed OR are adjusted for sex, age, BMI, metabolic syndrome, hepatic steatosis, alcohol consumption, intake of fast-food, vegetables, fruits, sweets, red and processed meat, white meat, fish, coffee and consumption of SSB. OR for Fib-4 score and Forns-Index were not adjusted for age since this variable is included in these indices.

Abbreviations: aOR–adjusted Odds ratio; NAFLD–non-alcoholic fatty liver disease; SSB–sugar-sweetened beverage; T2DM–type 2 diabetes mellitus; 95%CI– 95% confidence interval.

### Gender differences

In order to address gender differences with regard to nut consumption, we performed gender-specific subgroup analyses in males and females. Differences in baseline characteristics are displayed in S2 Table in S1 File. The negative association with NAFLD was even more pronounced in males consuming nuts ≥1time/day (aOR: 0.589 [95%CI: 0.411–0.844], p = 0.004). However, it could not be confirmed in female patients (aOR: 0.886 [95%CI: 0.616–1.275], p = 0.516; S3 Table in S1 File). Although a trend for decreased odds of having advanced fibrosis as assessed by Fib-4 score >2.67 was evident both in males and females consuming nuts 1–6 times/week, a significant associated could only be confirmed in males applying the Forns-index >6.9 (aOR: 0.610 [95%CI: 0.402–0.924], p = 0.020). Again, the adopted AHEI-2010 was not associated with NAFLD in males (aOR: 0.992, [95%CI: 0.982–1.003], p = 0.156) or females (aOR: 0.994, [95%CI: 0.982–1.007], p = 0.347).

## Discussion

Nuts have previously been associated with a reduced risk for MetS, especially T2DM, hypertension and obesity [11–14], and reduced risk for cardiovascular disease and death [33], while recently, a potentially protective effect on the prevalence of NAFLD was proposed in two Asian cohorts [19, 20]. In our study, we show that nut consumption at least once a day was associated with a significantly decreased risk for NAFLD while any consumption of nuts was linked to a lower prevalence of advanced fibrosis in NAFLD subjects. Moreover, we confirm other study results showing a lower prevalence of MetS and cardiovascular diseases among nut-consumers [13, 14].

Generally speaking, nuts are rich in monounsaturated and polyunsaturated fatty acids (PUFA), vegetable protein, fibers, folate, vitamins (e.g. tocopherols) and minerals (e.g. copper), among other bioactive compounds including carotenoids, phytosterols and polyphenolic compounds including flavonoids [5, 34–36]. Specifically, folate has been associated with counteracting atherothrombotic properties of homocysteine [37] while phytosterols might interfere with cholesterol absorption [4]. Tocopherol (i.e. vitamin E) and polyphenols have protective properties against free radicals and oxidative stress [4, 38], with flavonoid intake being linked to reduced all-cause, cardiovascular and cancer mortality [39], and tocopherol being widely used off-label to treat NAFLD due to its beneficial effects on liver enzyme elevations and histology improvement [40]. Other important components are omega-3-fatty-acids: Their dietary supplementation has recently been associated with reduced triglyceride levels and cardioprotective effects [41, 42]. Carotenoids—especially β-carotene–have also been attributed certain antioxidative effects, and recent study results showed that consuming high amounts of carotenoids lowered the risk of NAFLD [43], and improved lipid profiles in an animal models [44]. Finally, nondigestible components of nuts act as probiotics providing beneficial substrates for the gut microbiota [45]. Thus, several components and their synergetic interaction could explain a potential favorable effect on human physiology [4]. Interestingly, copper deficiency has previously been linked to disturbed iron homeostasis in NAFLD [46], potentially aggravating the natural course of NAFLD [47, 48]. Although copper supplementation by nut consumption might counteract harmful effects of copper deficiency in NAFLD, considerable variations in copper content of different nuts need to be considered.

Recently, Zhang et al [19] reported on a large retrospective cohort of 23915 patients excluding those with cardiovascular diseases or cancer, and found a significantly lower prevalence of NAFLD in patients consuming nuts ≥4 times/week, being equivalent to ≥200-240g/week. However, a smaller Chinese cohort study including 1068 patients, could only reproduce this finding in males within the highest quartile of nut intake [20]. Together, these studies add up to evidence from small cohort studies (<400 patients) reporting benefits for subjects with NAFLD [49–51]. Although we confirm these data in a representative cohort of Caucasian individuals, subgroup analyses could only reproduce this inverse association in male individuals. This is interesting since our findings go in line with previous studies reporting an association only in males [20, 49]. However, we can only speculate on potential explanations: First, the benefit of nut consumption might be better discernible in men on the background of more prevalent NAFLD and more pronounced MetS components [52]. Moreover, men usually have larger visceral fat mass, being associated with a more pro-inflammatory profile [53], which directly releases free-fatty acids into the portal vein, thereby promoting fat-accumulation in the liver [54]. Second, women are considered to have a higher awareness for healthy nutrition [55], which is also indicated by a higher prevalence of female gender in more frequent nut consumption groups in our study. Thus, the effect of nuts could be mitigated by other healthy dietary patterns.

To the best of our knowledge, this is the first study which proposes a beneficial effect of nuts on the prevalence of advanced fibrosis in patients with NAFLD. Although we cannot explain why the effect on fibrosis seems to start already when consuming nuts less frequently (i.e. 1–6 times/week), our data allow speculation that the beneficial effect on subsequent inflammation in the liver might already be evident at lower levels. Of note, we show that inflammation markers gradually decline with more frequent nut consumption, presumably indicating less inflammation, being the most important driver of fibrogenesis [56]. However, we acknowledge that blood inflammation markers may not fully mirror inflammation in the liver.

Additionally, it is unclear whether the beneficial effect of nuts follows a linear or U-shaped curve. Despite the large number of favorable bioactive compounds, nuts are also rich in fat and calories [4], consequently boosting one’s energy intake if consumed on a daily basis. This is supported by similar cholesterol and LDL-C levels among our patient groups independent from their amount of nut consumption. Thus, a frequent consumption might in some terms limit the beneficial effect of nuts’ healthy components.

This study has strengths with regard to investigating the link between fatty liver and dietary habits. To begin with, patients underwent careful metabolic and characterization in the absence of clinical suspicion or indication for diagnostic work-up. Due to the coincidence that endoscopies are mainly performed at the General Hospital Oberndorf in the local catchment area, we are confident that we could obtain a representative sample of the general population at risk for NAFLD (~60 years of age). Importantly, the fact that no other regulations existed on which patients were included in the study (e.g. type of insurance or comorbidities), mitigated selection bias, which is often introduced in other liver-centered studies.

However, several limitations need to be considered when interpreting our results. To begin with, we acknowledge that nut types are unique in their composition and their effect on human health [5] especially when prepared differently (e.g. roasted, cooked, salted). However, we did not specifically assess nut types and preparation. Thus, we cannot exclude that differences in preparation (e.g. with chocolate in candy or baked goods) mitigate the associations reported in this study, and might decrease the study’s impact. This is especially true for peanuts, which are commonly misclassified as nuts despite being legumes, which is likely to have occurred in our study. However, from the patient’s point-of-view, specifying different nut types would increase uncertainties on true consumption since their frequency again decreases, and overreporting. From a statistical point of view, it would decrease statistical power. Therefore, we could not dissect potentially diverse effects of different nut types. Secondly, dietary habits assessed using FFQ are always patient-reported and therefore subject to response and recall bias: The correctness of the answers given by the patients can never be verified, nor can the correct recall from the patients’ memory be verified. Also, FFQ require literacy and can be subject to overreporting [57].

Data from our study indicate that nuts are part of a healthier dietary pattern with significant differences among several healthy or unhealthy food types (Table 2). Demonstrated by a higher score in the AHEI-2020, these patients adhere to a healthier diet, which could accentuate the associations which were attributed to nut consumption. Nevertheless, the inverse association of nut consumption with NAFLD and advanced fibrosis remained significant after adjusting for several confounding food types such as red meat, coffee, and SSB, which have been linked to NAFLD previously [58–62]. Noteworthy, these significant dietary differences between patients consuming nuts and those who do not can also be observed in the majority of studies investigating the role of nuts [14], especially in those investigating NAFLD [19, 20].

Specific limitations of this study include the retrospective design which can only generate associations, but never causalities. Unfortunately, physical activity was only inconsistently reported and therefore could not be included as a covariate in regression models. Finally, NAFLD was diagnosed by abdominal ultrasound in our study. This is a limitation since hepatic steatosis can only be graded qualitatively, with particular weakness for lower degrees of liver fat. Therefore, a modest effect of any nut consumption might not be detected as a resolution of hepatic steatosis in our study.

Nevertheless, we demonstrate an inverse association of nut consumption with the prevalence of NAFLD in a large European cohort. In line with the literature, we confirm a stronger association in male gender. Moreover, we are the first to report a potentially favorable effect on the prevalence of advanced fibrosis. Our findings support recommendations for regular nut consumption as part of a Mediterranean diet, especially in patients with NAFLD [63]. Future dietary interventional studies investigating lipid-lowering and anti-inflammatory properties of nuts in patients with NAFLD are needed to investigate the full potential of this unique food type as an add-on treatment of NAFLD.

## Supporting information

S1 File (DOCX)

## Abbreviations

BMI: body mass index
BP: blood pressure
CCS: chronic coronary syndrome
FFQ: food frequency questionnaire
HDL-C: high-density lipoprotein cholesterol
HOMA-IR: Homeostasis Model Assessment for Insulin Resistance
IFG: impaired fasting glucose
IGT: impaired glucose tolerance
IQR: interquartile range
IR: insulin resistance
LDL-C: low-density lipoprotein cholesterol
MetS: metabolic syndrome
NAFLD: non-alcoholic fatty liver disease
NFS: NAFLD fibrosis score
OGTT: oral glucose tolerance test
PAD: peripheral artery disease
SSB: sugar-sweetened beverage
T2DM: type 2 diabetes mellitus
WC: waist circumference
