# Supplementary material for: Nut consumption and the prevalence and severity of non-alcoholic fatty liver disease
Source: PLoS One. 2020 Dec 31;15(12):e0244514. doi: 10.1371/journal.pone.0244514 (PMC7774938; doi:10.1371/journal.pone.0244514)
Supplement: S1 File — (DOCX) [file pone.0244514.s001.docx]

**S1 Table.** Markers of inflammation compared among patients stratified according to their nut consumption.

| **Patient characteristics** | **<1 time/week, n=2502** | **1-6 times/week, n=1506** | **1 time/day, n=505** | **≥2 times/day, n=142** | ***P* value** |
| --- | --- | --- | --- | --- | --- |
| Leukocytes, G/l | 6.2±1.7 | 6.1±1.7 | 5.5±1.4 | 5.7±1.3 | **<0.001** |
| C-reactive protein, mg/dl | 0.20 (0.10-0.40) | 0.17 (0.10-0.31) | 0.15 (0.08-0.30) | 0.11 (0.08-0.27) | **<0.001** |
| Erythrocyte sedimentation rate, mm | 6 (3-10) | 5 (2-9) | 6 (3-9) | 5 (2-9) | **0.001** |
| Ferritin, µg/l | 134 (71-231) | 114 (63-193) | 103 (54-178) | 89 (42-159) | **<0.001** |

**S2 Table.** Important patient characteristics in (**A**) male and **(B**) female patients compared among patients grouped according to their frequency of nut consumption.

| **A** | **All males,**  **n=2395** | **<1 time/week, n=1360** | **1-6 times/week, n=766** | **1 time/day, n=209** | **≥2 times/day, n=60** | ***P* value** |
| --- | --- | --- | --- | --- | --- | --- |
| Age, years | 58.3±9.8 | 58.5±10.1 | 57.3±9.3 | 59.8±8.6 | 59.1±9.4 | **0.004** |
| BMI, kg/mg² | 27.6±4.1 | 27.9±4.2 | 27.3±3.9 | 26.8±3.4 | 26.9±4.0 | **<0.001** |
| WC, cm* | 103±8 | 104±8 | 103±7 | 102±7 | 103±7 | **0.004** |
| Metabolic syndrome | 1225 (51.1%) | 743 (54.6%) | 361 (47.1%) | 93 (44.5%) | 28 (46.7%) | **0.001** |
| Hypertension | 1339 (55.9%) | 797 (58.6%) | 393 (51.3%) | 112 (53.6%) | 37 (61.7%) | **0.008** |
| T2DM/prediabetes | 1328 (55.7%) | 793 (58.7%) | 395 (51.6%) | 109 (52.2%) | 31 (52.5%) | **0.010** |
| T2DM | 385 (16.1%) | 255 (18.8%) | 91 (11.9%) | 25 (12.0%) | 14 (23.3%) | **<0.001** |
| Prediabetes | 943 (39.6%) | 538 (39.8%) | 304 (39.7%) | 84 (40.2%) | 17 (28.8%) | 0.402 |
| Dyslipidemia | 1104 (46.1%) | 662 (48.7%) | 334 (43.6%) | 87 (41.6%) | 21 (35.0%) | **0.017** |
| CCS | 184 (7.7%) | 122 (9.1%) | 47 (6.1%) | 12 (5.8%) | 3 (5.0%) | 0.053 |
| PAD | 79 (3.3%) | 60 (4.4%) | 14 (1.8%) | 4 (1.9%) | 1 (1.7%) | 0**.006** |
| Stroke | 83 (3.5%) | 58 (4.3%) | 19 (2.5%) | 5 (2.4%) | 1 (1.7%) | 0.098 |
| NAFLD | 1239 (53.1%) | 756 (56.7%) | 373 (50.7%) | 82 (39.8%) | 28 (47.5%) | **<0.001** |
| **B** | **All females,**  **n=2260** | **<1 time/week, n=1142** | **1-6 times/week, n=740** | **1 time/day, n=296** | **≥2 times/day, n=82** | ***P* value** |
| Age, years | 58.8±9.7 | 59.1±10.4 | 58.0±9.1 | 59.8±8.8 | 57.6±8.3 | **0.008** |
| BMI, kg/mg² | 26.5±5.2 | 27.2±5.5 | 26.1±4.9 | 25.3±4.5 | 25.1±4.7 | **<0.001** |
| WC, cm* | 103±12 | 105±12 | 102±10 | 101±9 | 99±9 | **<0.001** |
| Metabolic syndrome | 833 (36.9%) | 476 (41.7%) | 244 (33.0%) | 96 (32.4%) | 17 (20.7%) | **<0.001** |
| Hypertension | 1063 (47.0%) | 576 (50.4%) | 329 (44.5%) | 124 (41.9%) | 34 (41.5%) | **0.010** |
| T2DM/prediabetes | 959 (42.6%) | 541 (47.6%) | 284 (38.6%) | 117 (39.5%) | 17 (20.7%) | **<0.001** |
| T2DM | 253 (11.2%) | 167 (14.6%) | 60 (8.1%) | 22 (7.4%) | 4 (4.9%) | **<0.001** |
| Prediabetes | 706 (31.4%) | 374 (32.9%) | 224 (30.5%) | 95 (32.1%) | 13 (15.9%) | **0.013** |
| Dyslipidemia | 750 (33.2%) | 421 (36.9%) | 219 (29.6%) | 87 (29.4%) | 23 (28.0%) | **0.003** |
| CCS | 96 (4.3%) | 66 (5.8%) | 20 (2.7%) | 8 (2.7%) | 2 (2.5%) | **0.004** |
| PAD | 73 (3.2%) | 46 (4.0%) | 16 (2.2%) | 9 (3.0%) | 2 (2.4%) | 0.160 |
| Stroke | 65 (2.9%) | 42 (3.7%) | 13 (1.8%) | 9 (3.0%) | 1 (1.2%) | 0.083 |
| NAFLD | 745 (33.4%) | 428 (37.9%) | 221 (30.5%) | 76 (25.9%) | 20 (24.4%) | **<0.001** |

Abbreviations: BMI – body mass index; CCS – chronic coronary syndrome; NAFLD – non-alcoholic fatty liver disease; PAD – peripheral artery disease; T2DM – type 2 diabetes mellitus; WC – waist circumference;

**S3 Table.** Odds ratio (OR) and 95% confidence interval (95%CI) for NAFLD and advance fibrosis among groups of nut-consumers using binary logistic regression analyses adjusted for potential confounders. Analyses were based on subgroups of male and female individuals.

|  | | **Males** | | | **Females** | | |
| --- | --- | --- | --- | --- | --- | --- | --- |
|  | | **aOR (95% CI)** | ***P* value** | ***P* value for linear trend** | **aOR (95% CI)** | ***P* value** | ***P* value for linear trend** |
| NAFLD | **<1 time/week** | reference | reference | **0.009** | reference | reference | 0.516 |
|  | **1-6 times/week** | 0.904 (0.710-1.150) | 0.410 |  | 0.956 (0.721-1.267) | 0.752 |  |
|  | **≥1 time/day** | 0.589 (0.411-0.844) | **0.004** |  | 0.886 (0.616-1.275) | 0.515 |  |
| Fib-4 score >2.67 | **<1 time/week** | reference | reference | 0.620 | reference | reference | **0.040** |
|  | **1-6 times/week** | 0.580 (0.325-1.034) | 0.065 |  | 0.445 (0.170-1.162) | 0.098 |  |
|  | **≥1 time/day** | 1.097 (0.553-2.175) | 0.792 |  | 0.289 (0.064-1.299) | 0.106 |  |
| Forns-Index >6.9 | **<1 time/week** | reference | reference | **0.007** | reference | reference | 0.215 |
|  | **1-6 times/week** | 0.610 (0.402-0.924) | **0.020** |  | 0.499 (0.204-1.224) | 0.129 |  |
|  | **≥1 time/day** | 0.508 (0.259-0.998) | **0.049** |  | 0.633 (0.226-1.773) | 0.384 |  |

Displayed OR are adjusted for sex, age, BMI, metabolic syndrome, hepatic steatosis, alcohol consumption, intake of fast-food, vegetables, fruits, sweets, red and processed meat, white meat, fish, coffee and consumption of SSB. OR for Fib-4 score and Forns-Index were not adjusted for age since this variable is included in these indices.

Abbreviations: aOR – adjusted Odds ratio; NAFLD – non-alcoholic fatty liver disease; SSB – sugar-sweetened beverage; T2DM – type 2 diabetes mellitus; 95%CI – 95% confidence interval

**REFERENCES**

1. Zhang S, Fu J, Zhang Q, et al. Association between nut consumption and non-alcoholic fatty liver disease in adults. *Liver international : official journal of the International Association for the Study of the Liver.* 2019;39(9):1732-1741.

2. Chen Bb, Han Y, Pan X, et al. Association between nut intake and non-alcoholic fatty liver disease risk: a retrospective case-control study in a sample of Chinese Han adults. *BMJ Open.* 2019;9(9):e028961.
